# Optimization of the tumor microenvironment and nanomedicine properties simultaneously to improve tumor therapy

**DOI:** 10.18632/oncotarget.11546

**Published:** 2016-08-23

**Authors:** Bo Zhang, Wei Shi, Ting Jiang, Lanting Wang, Heng Mei, Heng Lu, Yu Hu, Zhiqing Pang

**Affiliations:** ^1^ Institute of Hematology, Union Hospital, Tongji Medical College, Huazhong University of Science & Technology, Wuhan, Hubei, PR China; ^2^ Key Laboratory of Smart Drug Delivery, Ministry of Education, School of Pharmacy, Fudan University, Shanghai, China; ^3^ School of Medicine, Fudan University, Shanghai, China; ^4^ Collaborative Innovation Center of Hematology, Huazhong University of Science and Technology, China

**Keywords:** tumor microenvironment, imatinib mesylate, nanoparticles, micelles, nanomedicine size

## Abstract

Effective delivery of nanomedicines to tumor tissues depends on both the tumor microenvironment and nanomedicine properties. Accordingly, tumor microenvironment modification or advanced design of nanomedicine was emerging to improve nanomedicine delivery to tumors. However, few studies have emphasized the necessity to optimize the tumor microenvironment and nanomedicine properties simultaneously to improve tumor treatment. In the present study, imatinib mesylate (IMA) was used to normalize the tumor microenvironment including platelet-derived growth factor receptor-β expression inhibition, tumor vessel normalization, and tumor perfusion improvement as demonstrated by immunofluorescence staining. In addition, the effect of tumor microenvironment normalization on tumor delivery of nanomedicines with different sizes was carefully investigated. It was shown that IMA treatment significantly reduced the accumulation of nanoparticles (NPs) around 110 nm but enhanced the accumulation of micelles around 23 nm by *in vivo* fluorescence imaging experiment. Furthermore, IMA treatment limited the distribution of NPs inside tumors but increased that of micelles with a more homogeneous pattern. Finally, the anti-tumor efficacy study displayed that IMA pretreatment could significantly increase the therapeutic effects of paclitaxel-loaded micelles. All-together, a new strategy to improve nanomedicine delivery to tumor was provided by optimizing both nanomedicine size and the tumor microenvironment simultaneously, and it will have great potential in clinics for tumor treatment.

## INTRODUCTION

Nowadays nanomedicines have become the mainstream for tumor therapy [1], owning to their unique superiorities to small molecules [2]. The effective delivery of nanomedicines to tumor tissues depends on both nanomedicine properties and the tumor microenvironment including dense matrix, high interstitial fluid pressure (IFP), heterogeneous vascular distribution, and poor tumor perfusion [3, 4]. Robust evidences have shown that because of the complex tumor microenvironment, tumor delivery of nanomedicines was highly influenced by the physiochemical properties of the nanomedicines including size [5, 6], shape [7], charge [8, 9] and surface modification [10], among which particle size was very crucial in dominating the tumor delivery of nanomedicines. As previously reported, larger size (100 nm) favored the global accumulation of nanomedicines in tumors, while relative smaller size (10-30 nm) contributed to the effective penetration and homogeneous distribution of nanomedicines in tumor tissues [5, 11]. To achieve improved nanomedicine delivery to tumor tissues, both nanomedicine properties and the tumor microenvironment could be optimized. On the one hand, nanomedicines could be elaborately designed to achieve improved tumor delivery by the size shrink strategy [12, 13] or the tumor microenvironment-responsive strategy [14, 15]. However, these advanced nanomedicines still could not conquer the resistance of drug delivery from the complex tumor microenvironment [3]. On the other hand, strategies modifying the tumor microenvironment including tumor matrix disruption and tumor vessel normalization were also emerging to improve the delivery of nanomedicines for tumor treatment [16, 17]. To the best of our knowledge, few studies highlighted the necessity to optimize both nanomedicine properties and the tumor microenvironment simultaneously to achieve a pronounced improvement in tumor therapy.

Imatinib mesylate (IMA), a clinically approved drug for the treatment of chronic myeloid leukemia and gastrointestinal stromal tumor, has been successfully exploited to inhibit platelet-derived growth factor receptor-β (PDGF-β) signaling and reduce tumor IFP to improve small-molecule drug delivery for tumor therapy in animal models [18, 19]. However, the specific mechanisms associated with IFP reduction by inhibition of PDGF-β signaling such as the density of tumor vessels, pericyte coverage rate of endothelial cells, and tumor perfusion have not been investigated in detail in these studies.

**Figure 1:**
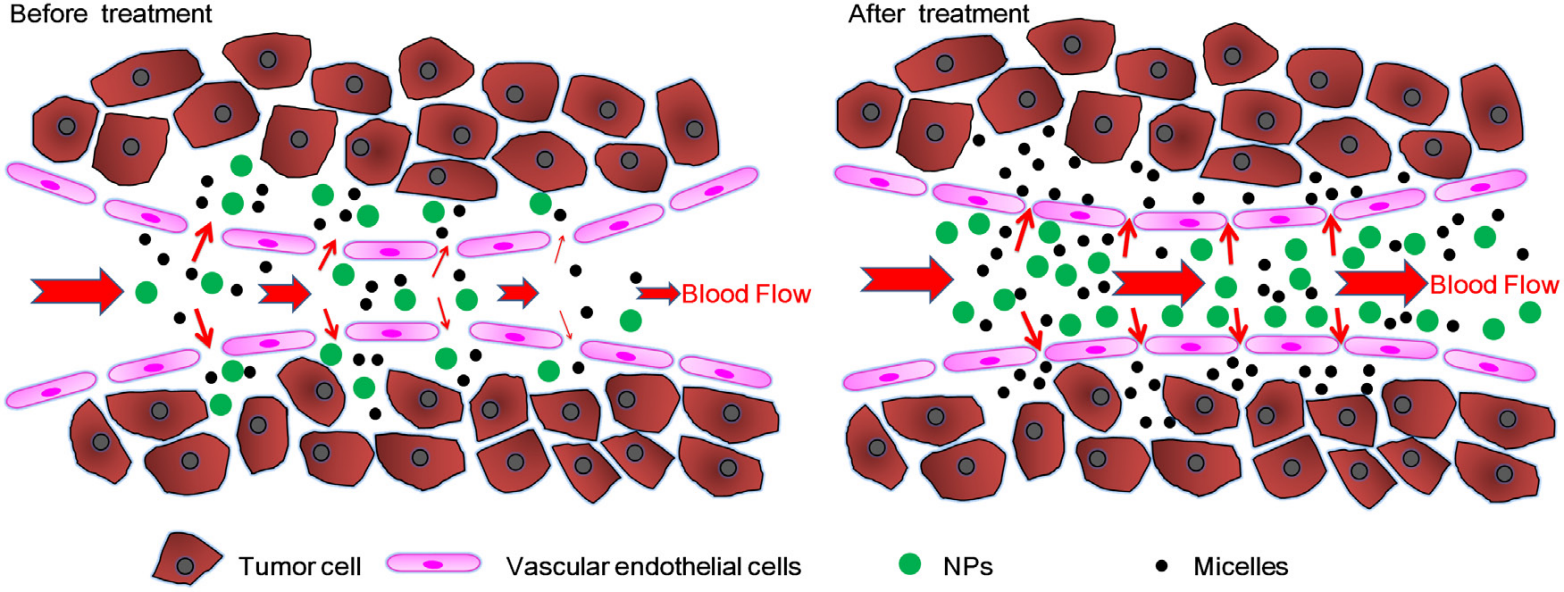
Schematic graph of tumor vessel normalization, tumor perfusion, and delivery of nanomedicines with different sizes to A549 tumors before and after IMA treatment

In the present study, a new strategy to improve nanomedicine delivery to tumors was provided by optimizing both nanomedicine properties and the tumor microenvironment simultaneously. Firstly, IMA was used to modify the tumor microenvironment and the effect of tumor microenvironment modification on *in vivo* delivery of nanomedicines with different size (Figure 1) was evaluated. Tumor microenvironment modification including PDGFR-β expression inhibition, tumor vessel densities, tumor vessel normalization, and tumor perfusion were assessed by immunofluorescence staining of tumor slices. Biodegradable block copolymer polyethylene glycol-polylactic acid (PEG-PLA)-based nanoparticles (NPs) around 110 nm and micelles around 23 nm were used as model nanomedicines with different size. The effect of tumor microenvironment modification on tumor delivery of nanomedicines with different size was investigated by both *in vivo* imaging and distribution experiments. Secondly, the classical chemotherapeutics paclitaxel (PTX)-loaded micelles was used as the model nanomedicine combining IMA pretreatment to perform the anti-tumor efficacy study. As far as we were concerned, it was the first report that emphasized the importance of optimizing nanomedicine size and the tumor microenvironment simultaneously to achieve an ideal therapeutic effect.

## RESULTS AND DISCUSSION

Size-dependent delivery of nanomedicines to tumor has attracted intense attention nowadays [5, 6], which not only occurred to primary tumor [11], but also occurred to the tumor metastasis site [20]. As for primary tumor, the size-dependent effect always appeared in poorly permeable tumors with relative more ECM, but it was not so obvious in highly permeable tumors with relative more tumor vessels [21]. As far as we were concerned, few studies stressed the size-dependent effect following tumor microenvironment modification and emphasized the importance of optimizing the nanomedicine size and the tumor microenvironment simultaneously to achieve improved therapeutic benefits. To demonstrate the idea, A549 lung cancer with a certain amount of both tumor vessels and ECM [16, 22] was selected as the tumor model, and IMA widely used in clinics [23, 24] was utilized as the tumor microenvironment modifier in the present study.

As shown in Supplementary Figure S1, after three weeks of IMA treatment at the daily dose 50 mg/kg ended, it did not exert significant differences in both tumor volume and body weight of animal models as compared with deionized water treatment. These results agreed with other tumor microenvironment modifiers as previously documented including losartan, chloroquine, rapamycin, etc [17, 25, 26], indicating the dose of IMA used in the present study might be safe for animal models without significant therapeutic effects or adverse responses.

**Figure 2:**
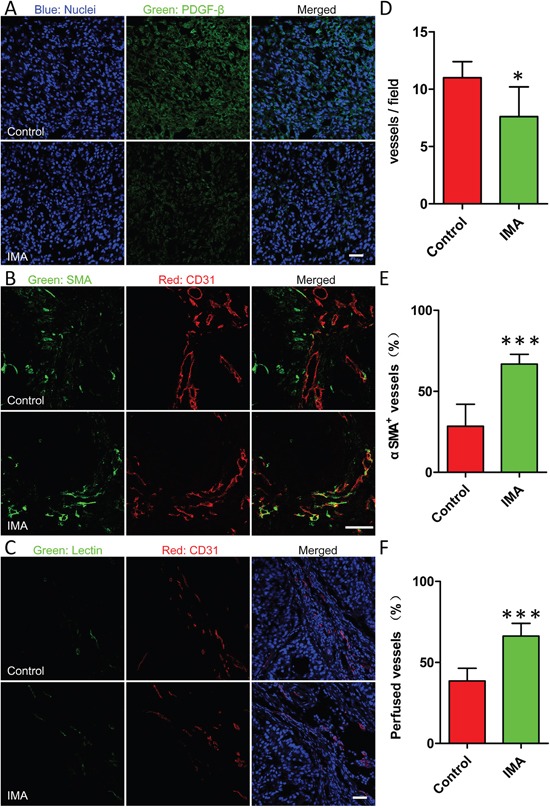
Effects of IMA treatment on the tumor microenvironment including **A.** reduction of PDGFR-β expression in tumor tissues, **B.** normalization of tumor vessels, and **C.** improvement of tumor perfusion. The changes of **D.** the density of vessels, **E.** coverage rate of pericyte on endothelial and **F.** the percentage of lectin-labeled vessels before and after IMA treatment. ^*^P<0.05, ^***^ *P*<0.001 compared with the control group. After A549 xenograft-bearing mouse models were treated with 50 mg/kg of IMA or equal volume of water for three weeks, mouse models were sacrificed and tumor xenografts were obtained for frozen tumor slices preparation and immunofluorescence staining. Functional blood vessels were identified by green staining after i.v. injection of 5 mg/kg of DyLight^®^ 488-labeled lectin to A549 xenograft-bearing mouse models, and co-localization of the CD31 and DyLight^®^ 488-lectin signals in the tumor sections were analyzed using the Image J software to evaluate tumor perfusion. The bar indicated 50 μm.

Subsequently, tumor microenvironment modification by IMA treatment was investigated by immunofluorescence staining (Figure 2). The results demonstrated that IMA treatment at a daily dose of 50 mg/kg for three weeks could significantly inhibited PDGFR-β expression level (Figure 2A), consisted well to prior reports [27]. It has been documented that inhibiting PDGFR-β expression could cause tumor vessel regression by stripping out pericytes in some tumor vessels while normalize other tumor vessels [27]. The fraction of vessel regression to vessel normalization might be associated closely with tumor types, the dose and the administration route as well as the type of PDGFR-β inhibitors [18, 23, 28, 29]. According to previous reports, in some tumor types, such as melanoma [24] and colon cancer [28], tumor vessel regression outweighed tumor vessel normalization. The pore size between tumor vascular endothelial cells in these tumors was enlarged on the whole, which could improve the enhanced permeability and retention (EPR) effect and thus the delivery of nanomedicine around 110 nm [24]. When tumor vessel regression went too far beyond tumor vessel normalization, the tumor xenograft growth might be severely suppressed, and then therapeutic benefits rather than tumor microenvironment modification was obtained [29]. As a comparison, in lung cancer [27, 30], tumor vessel normalization outweighed tumor vessel regression, which reduced the pore size between tumor vascular endothelial cells and mainly improved small-molecule drug delivery [30].

Previously, IMA was used to inhibit PDGF-β signaling pathway to lower IFP in tumor tissues to improve the delivery of free small-molecule drugs [19, 27]. However, the definite mechanism of IFP reduction was not clarified clearly. In the present study, tumor microenvironment parameters including the density, the structure, and the functionality of tumor vessels were investigated in details. As shown in Figure 2, the density of CD31-labeled tumor vessels in the IMA treatment group was reduced, about 69.1% as compared with that of control group (Figure 2D). However, the percentage of endothelial cells covered by SMA-labeled pericytes was increased from 28.4 ± 13.6% in the control group to 66.8 ± 6.0% in the IMA treatment group, indicating IMA treatment significantly improved tumor vessel normalization of A549 xenografts (Figure 2B and 2E). Accordingly, the percentage of functional vessels, indicated by tumor perfusion experiment, was also markedly improved, increasing from 38.6 ± 7.7% in the control group to 66.2 ± 7.9% in the IMA treatment group (Figure 2C and 2F). As the functional properties of tumor vessels always outweighed the density of tumor vessels [27], the normalization of tumor vessels would offset the reduction of vessel density and thus improve tumor perfusion. As PDGF-β signaling pathway was also involved in tumor ECM production [31], inhibiting PDGF-β pathway would disrupt ECM to a certain degree, which could decompress vessels and also contribute to tumor perfusion improvement [32]. Overall, IMA treatment in our study helped normalize tumor microenvironment and improve tumor perfusion successfully.

**Figure 3:**
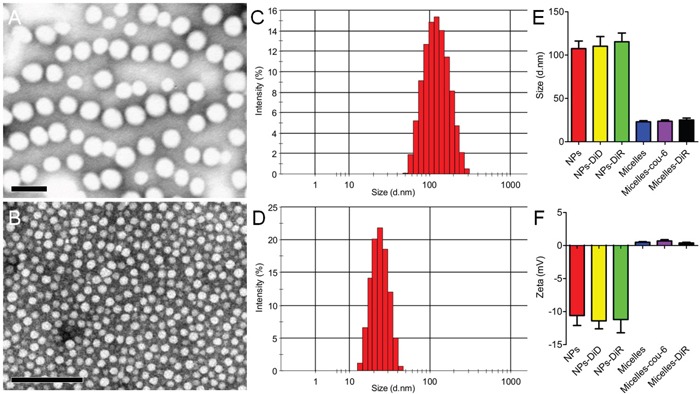
Characterizations of nanomedicines including NPs A, C, E, F. and micelles B, D, E, F TEM photograph of (A) NPs and (B) micelles stained with 2% phosphotungstic acid. Bar: 100 nm. The size distribution of (C) NPs and (D) micelles analyzed with a Malvern Nano ZS. (E) Size and (F) Zeta potential of different types of NPs and micelles (n=3).

**Figure 4:**
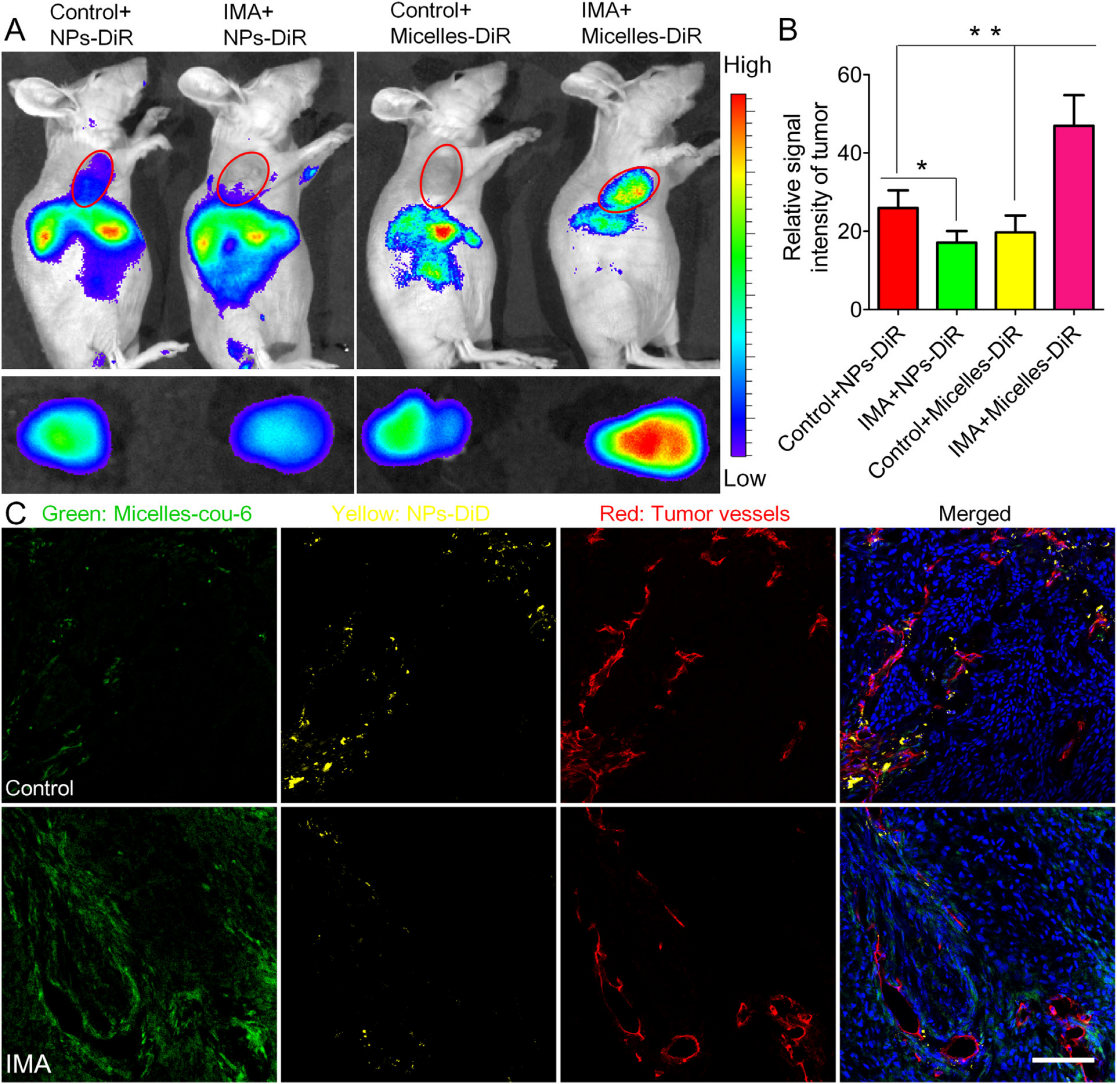
The effects of IMA treatment on tumor nanomedicine delivery **A.** *In vivo* fluorescence imaging of A549 xenograft-bearing mice (the upper row) treated with IMA or deionized water, *ex vivo* fluorescence imaging of their corresponding tumor xenografts (the lower row), and **B.** the relative signal intensity of tumor tissue 24 h post the injection of NPs-DiR or micelles-DiR. ^*^ *P*<0.05, compared with the Control+NPs-DiR group. ^**^ *P*<0.01 compared with the IMA+Micelles-DiR group. **C.** *In vivo* distribution of micelles-cou-6 and NPs-DiD in tumor slices from A549 tumor xenograft-bearing mouse models treated with IMA or deionized water at 24 h after i.v. injection of a mixture of micelles-cou-6 and NPs-DiD. The oral dose of IMA was 50 mg/kg for three weeks. The dose of both coumarin-6 and DiD was 0.05 mg/kg. The bar indicated 100 μm.

To investigate the necessity of optimizing nanomedicine size simultaneously, nanomedicines including smaller micelles around 23 nm and larger NPs around 110 nm based on biodegradable materials MPEG-PLA were designed. Both micelles and NPs showed a favorable polydispersity index (PDI) and a narrow distribution (Figure 3C and 3D). TEM photographs showed both blank micelles and NPs were of regular size and smooth surface (Figure 3A and 3B), in good agreement with the requirements of *in vivo* experiments as reported [33]. As the loading mass of fluorescence trackers in micelles or NPs was minimal, the encapsulation process did not influence their particle size and Zeta potential significantly (Figure 3E and 3F). The DLC of Coumarin-6 and DiR in micelles was 0.082 ± 0.005% and 0.627 ± 0.057%, respectively. The DLC of DiD and DiR in NPs was 0.064 ± 0.003% and 0.421 ± 0.049%, respectively. Furthermore, the cumulative release of fluorescence trackers from nanomedicines including micelles and NPs was less than 1% during 24 h in PBS (pH=7.4, 0.01M) and 5% mouse serum. These results agreed well to previous reports [34, 35] and indicated that the signal of coumain-6, DiD or DiR could favorably represent the behavior of micelles or NPs in *in vivo* experiment. As shown in Figure 4, *In vivo* fluorescence imaging experiments demonstrated that pretreatment with IMA for three weeks could increase the accumulation of 23 nm-sized Micelles-DiR in tumor tissues (Figure 4A and 4B), with the fluorescence signal intensity in the tumor site 2.38 fold higher than that of the control group. However, pretreatment with IMA decreased the accumulation of 110 nm-sized NPs in tumor tissues, with the fluorescence signal intensity in the tumor site only 65.9 % that of the control group (Figure 4A and 4B). *Ex vivo* imaging of major organs showed that there were no significant differences in the fluorescence intensity of livers, hearts, lungs and brains among these four groups (Supplementary Figure S2). The IMA pretreatment could increase NPs accumulation but exert no effect on micelles accumulation in spleens. The results might be due to IMA-induced expansion of myeloid cells in spleen [36], which could uptake more NPs around 100 nm than smaller micelles [37]. In addition, fluorescence signal of micelles in kidneys was much stronger than that of NPs, with no association with IMA treatment, which was largely due to the small size of micelles [37, 38].

Consistent to the accumulation displayed in the *in vivo* imaging experiment, the *in vivo* nanomedicine distribution experiments (Figure 4C) demonstrated that in the IMA pretreatment group, small micelles could distribute more extensively in the tumor tissues than its counterpart control group, even those regions far away from tumor vessels. As for the larger NPs, IMA pretreatment inversely compromised its distribution. In addition, larger NPs were mainly located near tumor vessels [13]. The reasons of these outcomes could be explained as follows: Firstly, IMA treatment normalized most tumor vessels that did not regress. As functional property was much more important in tumor perfusion than the density of vessels did, the global tumor perfusion was increased after IMA pretreatment and more micelles and NPs might be transported to the tumor site. Secondly, the pore size between endothelial cells of normalized tumor vessels was prominently reduced, which decreased the permeability of larger NPs but exerted no significant influence on smaller micelles or free small-molecule drugs [39]. When nanomedicines in the tumor vessels were removed by heart perfusion, a favorable distribution of micelles and a compromised distribution of NPs were observed. The results again verified the importance to optimize the tumor microenvironment and nanomedicine properties simultaneously to achieve tumor therapy improvement [3].

**Figure 5:**
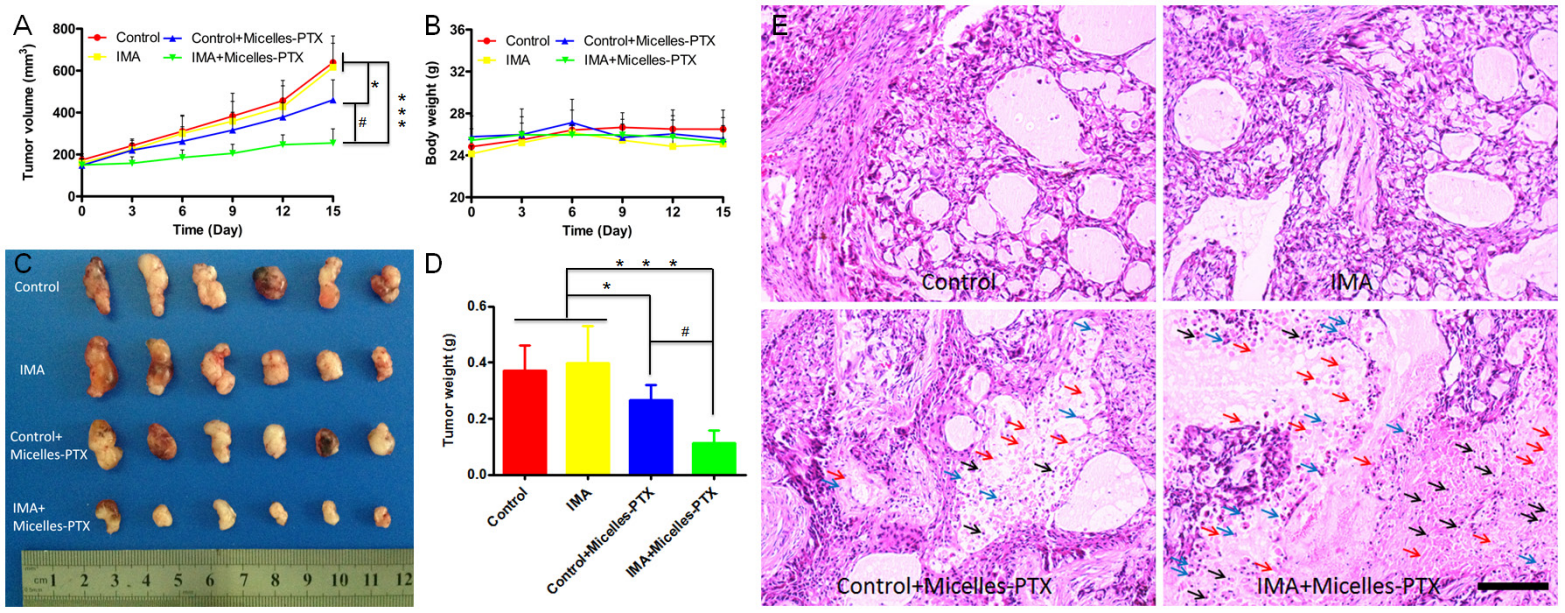
Micelles-PTX combined with IMA pretreatment significantly inhibited the growth of tumors **A.** Tumor growth curve and **B.** mouse body weight curve throughout the experiment. **C.** Tumor xenografts images and **D.** tumor weights at the study end point. ^*^ *P*<0.05, ^***^ *P*<0.001 compared with the control or IMA group, ^#^ *P*<0.01 compared with the Control+Micelles-PTX group. **E.** H & E staining of A549 xenograft slices from mice after various treatments. Black, blue and red arrows indicated karyopyknosis, karyorrhexis and karyolysis, respectively. The bar indicated 100 μm. Mouse models with size-matched A549 tumor xenografts were randomly assigned into four groups (n=6) and received oral treatment of IMA (50 mg/kg) or deionized water for two weeks followed by Micelles-PTX treatment. After Micelles-PTX treatment started, IMA or deionized water treatment was continued for another week. Micelles-PTX treatment was continued every third day for five times with the PTX dose of 5 mg/kg.

Based on the results of the *in vivo* imaging and distribution experiments, smaller micelles were selected as the model nanomedicine and PTX with favorable hydrophobicity as the model therapeutics to perform the anti-tumor efficacy study. As shown in Supplementary Table S1, PTX loading to micelles slightly increased their particle size and PDI, and slightly decreased their zeta potential. The DLC and EE of PTX in micelles were 13.0 ± 0.6% and 90.3 ± 3.4 %, respectively. The releasing of PTX from Micelles-PTX was much more slowly than commercial Taxol (Supplementary Figure S3), in good agreement with previous studies [33, 40]. Results revealed tumor xenografts in both the control group and the IMA group grew rapidly and there was no significant difference in the tumor size between these two groups (Figure 5A). The results consisted well to previous studies where IMA was sued to modify the tumor microenvironment to improve *in vivo* delivery of free drugs to tumors [27]. Micelles-PTX treatment without IMA pretreatment obtained only modest therapeutic benefits, mainly due to the heterogeneous EPR effect [41]. As compared with the control group, the *TGIR_v_* and *TGIR_w_* of the control+Micelles-PTX group were 28.0% and 23.9%, respectively (Figure 5A, 5C and 5D). As a comparison, when compared with the control group, Micelles-PTX treatment pretreated with IMA achieved the most significant shrinkage of tumor size. The *TGIR_v_* and *TGIR_w_* of the IMA+Micelles-PTX group were 60.1% and 63.4%, respectively (Figure 5A, 5C and 5D), which was consistent to the *in vivo* imaging and distribution experiments. H & E staining of tumor slice further verified the findings of tumor growth inhibition experiment. Morphology changes of nuclear such as karyopyknosis, karyorrhexis and karyolysis were negligible in both control and IMA treatment groups. As a comparison, more obvious changes were displayed in PTX treated groups, in which a much more extensive tumor necrosis including karyopyknosis, karyorrhexis and karyolysis was found in Micelles-PTX pretreated with IMA group (Figure 5E). In addition, Micelles-PTX pretreated with IMA did not reduce the body weight of mouse models during the experiment (Figure 5B), indicating this treatment might be safe. Altogether, the anti-tumor efficacy study demonstrated that Micelles-PTX pretreated with IMA resulted in the strongest anti-tumor activity.

Recently, researches focused on breaching drug delivery barriers presented by the tumor microenvironment by normalizing the tumor microenvironment, including tumor vessel normalization for tumors rich in vascularity and tumor matrix disruption for tumor highly desmoplastic [16]. However, the tumor microenvironment modifiers always have complicated effects on normalizing the tumor microenvironment in different tumor types. For instance, IMA could normalize tumor vessels in lung cancer [27, 30], but cause tumor vessel regression in colon cancer [18]. Therefore, to improve nanomedicine delivery to tumor tissues, it is necessary to integrate tumor microenvironment normalization strategy and nanomedicine property. However, few studies have emphasized the necessity to optimize nanomedicine properties according to the tumor microenvironment normalization strategy. As tumor vessel normalization would decrease the pore size between endothelial cells, it would preferentially benefit the *in vivo* delivery of smaller nanomedicines rather than larger nanomedicines [39] as displayed in the present study. As a comparison, tumor matrix disruption not only alleviated the mechanical force to decompress tumor vessels, increased tumor perfusion, and enhanced drug delivery to tumor tissue [32], but also reduced the penetrating resistance of tumor matrix to nanomedicines and favored a more homogeneous distribution pattern of nanomedicines regardless of size [3, 17]. In addition, tumor microenvironment normalization not only improved drug delivery for primary tumors, but also might relieve tumor metastasis burden. Specially, tumor vessel normalization could reduce the shedding of tumor cells into the vascular system [42], which was considered as a prerequisite for tumor metastasis. Tumor matrix disruption could also reduce the occurrence of tumor metastasis by reducing matrix component such as fibrin and hyaluronic acid [43, 44], which was crucial for the implant of tumor cells in normal organs. Furthermore, it was indeed not easy to judge the endpoint of the tumor microenvironment normalization. The efficacy of IMA treatment in improving tumor nanomedicine delivery is an important criterion. In previous report, IMA was orally administrated at the dose of 150 mg/kg for 4 weeks to improve tumor delivery of small-molecule chemotherapeutics [27]. In the present study, IMA treatment was initiated at a much lower dose 50mg/kg to avoid adverse effect to animal models. To optimize the duration time of IMA treatment, the effect of IMA treatment time on tumor nanomedicine delivery was investigated by *in vivo* imaging using Micelles-DiR as the model nanomedicine. The results demonstrated that after two-week and three-week IMA treatment, micelles accumulation in tumor was increased to 1.4-fold (Supplementary Figure S4) and 2.38-fold (Figure 4A and 4B) of that of control, respectively, which directly reflected that three-week IMA treatment had higher efficacy in improving smaller nanomedicine delivery to tumor, and thus IMA treatment at a dose of 50mg/kg for three weeks was used throughout our study. Altogether, when judicious dose of tumor microenvironment modifier was rationally used, the tumor microenvironment normalization strategy could be effectively and safely applied when combined with nanomedicines with a suitable size.

## MATERIALS AND METHODS

IMA was from Dalian Meilun Biotech Co., Ltd (Dalian, China). DyLight^®^ 488-labeled tomato lectin (Lycopersicon esculentum) was ordered from Vector (USA). Cy™ 3-conjugated SMA-α mouse monoclonal primary antibody and fluorescence tracker coumarin-6 were purchased from sigma (USA). Hoechst 33342 was from Beyotime^®^ Biotechnology Co., Ltd. (Nantong, China). A near-infrared dye, 1,1′-Dioctadecyl-3,3,3′,3′-tetramethylindo-tricarbocyanineiodide (DiR) and 4-chlorobenzenesulfonate salt (DiD) dye were bought from Invitrogen (USA). PDGFR-β rabbit polyclonal primary antibody was from Santa Cruz biotechnology (USA). CD31 goat polyclonal primary antibody was from R&D (USA). Alexa Fluor^®^ 488-conjugated donkey-goat secondary antibody and Alexa Fluor^®^ 488-conjugated donkey-rabbit secondary antibody were from Jackson (USA). Normal mouse serum was from Yeasen Biotech (Shanghai, China). MPEG_2000_-PLA_2000_ was bought from Jinan Daigang Biomaterial Co., Ltd (Jinan, China). Methoxy-PEG (MPEG, MW 3000 Da) was purchased from NOF (Tokyo, Japan) and D, L-lactide (purity: 99.5%) was ordered from PURAC (Arkelsedijk, Holland). Methoxy-poly (ethylene glycol)-poly (lactic acid) (MPEG–PLA, Mw 33000 Da) block copolymers were synthesized by ring-opening polymerization of D, L-lactide using MPEG as the initiator as described previously [45]. Sodium cholate was from Shanghai Chemical (Shanghai, China). Foetal bovine serum (FBS), trypsin-EDTA (0.25%), cell culture medium and penicillin-streptomycin were bought from Gibco (CA). Deionised water from the Millipore Simplicity System (Millipore, Bedford, MA) was used in all experiments. All other reagents were of analytical reagent grade and were from Sinopharm Chemical Reagent (Shanghai, China).

### Cells and animal models

A549 cell lines were from the Chinese Academy of Sciences Cell Bank (Shanghai, China) and maintained in DMEM supplemented with 10% FBS and 100 U/ml penicillin/streptomycin at 37°C with 5% CO_2_ atmosphere. Male Balb/c nude mice aged eight weeks were from the Shanghai Slac Lab Animal Ltd. (Shanghai, China) and used according to the ethics committee of Fudan University. To establish tumor xenograft-bearing mouse models, a cell suspension of A549 tumor cells (5×10^6^ cells in 100 μl of PBS) was injected subcutaneously into nude mice. Tumor diameters were measured with a caliper and tumor volumes were determined by the formula: Tumor volume (mm^3^) = 0.5 × d_max_ × d_min_^2^, where d_max_ represented the maximum diameter and d_min_ represented its perpendicular diameter. Mouse models with tumor xenografts around 4 mm in diameter were selected for subsequent IMA treatment.

### IMA treatment

IMA was dissolved in deionized water with the concentration of 10 mg/ml and orally administrated to mouse models by gauge once every day at the dose of 50 mg/kg for three weeks. Mouse models receiving deionized water of equal volume served as control.

### Tumor microenvironment modification

After three weeks of IMA treatment, mouse models were sacrificed and perfused with 4% paraformaldehyde, and then tumor xenografts were obtained for frozen tumor slices preparation, immunofluorescence staining, and observation under a confocal microscope (ZEISS, 710, LSM, Germany). Immunofluorescence staining of tumor slices was performed as described elsewhere [46]. In brief, tumor sections were pre-blocked with 10% goat serum at room temperature for 1 h, incubated with primary antibody at 4°C overnight, and then labeled with the corresponding secondary antibody for 1 h. For PDGFR-β staining, primary rabbit polyclonal PDGFR-β antibody (1:100) and Alexa Fluor^®^488-conjugated donkey-rabbit secondary antibody (1:100) were used. To observe the changes of vascular structure, pericytes were labeled with Cy™ 3-conjugated SMA-α mouse monoclonal primary antibody (1:200). Vascular endothelial cells were labeled with CD31 goat polyclonal primary antibody (1:100) and Alexa Fluor^®^ 488-conjugated donkey-goat secondary antibody (1:100). To assess tumor vessel density, the number of CD 31-labeled vessels per field of vision were calculated in six randomly-assigned regions of each tumor under the confocal microscope and compared with that of the control group (n=5). CD31-positive blood vessels covered by pericytes were generally identified as normalized blood vessels [25]. To assess tumor vessel normalization, the percentage of tumor vessels covered by pericytes was calculated in six randomly-assigned regions in each tumor under the confocal microscope (n=5). For tumor perfusion assessment, after IMA treatment ended, mouse models received i.v. administration of DyLight^®^ 488-labeled tomato lectin at the dose of 5 mg/kg followed by heart-perfusion 1 h post injection, and then tumor xenografts were sliced for CD 31 staining as described above. Tumor slices were observed under a confocal microscopy (ZEISS, 710, LSM, Germany). CD31-positive blood vessels that co-localized with DyLight^®^ 488-lectin were identified as well perfused blood vessels [25], and tumor perfusion was indicated as the percentage of well perfused vessels in all tumor vessels. To assess the tumor perfusion, the co-localization of the CD31 and DyLight^®^ 488-lectin signals (perfused vessels) in the tumor sections were captured in six randomly-assigned regions in each tumor (n=5) and further analyzed using the Image J software.

### Preparation and characterization of nanomedicines

NPs based on MPEG-PLA were prepared by an emulsion and solvent evaporation method as previously described [33]. Briefly, 30 mg of MPEG-PLA (Mw=33000 Da) was dissolved in 1 ml of dichloromethane. Then, the polymer solution was added into 5 ml of 0.6% sodium cholate aqueous solution and sonicated (200 W, 5 s for 15 times) in an ice bath by a probe sonicator (Scientz Biotechnology Co. Ltd., China). After removing dichloromethane by a ZX-98 rotary evaporator (Shanghai Institute of Organic Chemistry, China), NPs were collected by centrifugation with a TJ -25 centrifuge (Beckman Counter, USA), and then re-suspended in 2 ml of PBS (0.01 M, pH=7.4). Micelles based on MPEG-PLA were developed by a thin-film hydration technique as previously described [47]. In brief, 30 mg of MPEG_2000_-PLA_2000_ was dissolved in 3 ml of acetonitrile, and the polymer solution was evaporated for 2 h at 40°C with a ZX-98 rotary evaporator (Shanghai Institute of Organic Chemistry, China) to remove acetonitrile. The thin polymeric film in the round-bottom flask was hydrated with 2 ml of PBS (0.01 M, pH=7.4) and a micelle solution was acquired. Fluorescence trackers coumarin-6 and DiR was used to label micelles and DiD and DiR was used to label NPs. Chemotherapeutics PTX was encapsulated into micelles for the anti-tumor efficacy study. These types of nanomedicines were developed with the same process as blank nanomedicines except that 30 μg of coumarin-6, 30 μg of DiD, 200 μg of DiR or 5 mg of PTX was added into the polymer solution in advance. Nanomedicines were subjected to a 1.6×20 cm sepharose CL-4B column eluted with PBS to remove free fluorescence trackers or PTX.

Particle size and zeta potential of different types of NPs and micelles were analyzed using a Malvern Nano ZS (Malvern Instruments, UK). The morphology of blank NPs and micelles were detected under a transmission electron microscope (TEM) (H-600, Hitachi, Japan) following negative staining with 2% phosphotungstic acid.

For the determination of drug loading capacity (DLC) and encapsulation efficiency (EE), nanomedicines were dissolved by acetonitrile and then the fluorescence tracker or therapeutics was measured. Coumarin-6 and PTX were quantitatively determined by a high performance liquid chromatography (HPLC) method, and DiD and DiR were quantitatively analyzed by a microplate reader as described previously [35, 40, 48]. DLC and EE were calculated by the formulas: $\text{DLC}=\frac{\text{Drug encapsulated}}{\text{Total nanomedicine}},\text{EE}=\frac{\text{Drug encapsulated}}{\text{Drug imput}}$.

The dialysis method was performed to evaluate the release of DiD and DiR from NPs, and coumarin-6 and DiR from micelles by using PBS (pH=7.4, 0.01 M) with 0.5% Tween-80 and 5% percent murine serum in PBS (pH=7.4, 0.01 M) as the release medium as previously reported [35, 40]. The dialysis bags containing 1 mg of fluorescence tracker in 1 mL of release medium were incubated in 10 mL of the same release medium at 37°C with shaking at 100 rpm/min. After sampling at predetermined time points, equal volumes of fresh release medium were added. The concentration of fluorescence tracker including coumarin-6, DiD and DiR were quantitative analyzed as mentioned above.

### *In vivo* imaging

After three weeks of IMA treatment, tumor xenograft-bearing mice were i.v. injected with DiR-labeled NPs (NPs-DiR) or micelles (Micelles-DiR) at the DiR dose of 0.5 mg/kg. 24 h later, mouse models were subjected to *in vivo* imaging using the *In Vivo* IVIS spectrum imaging system (PerkinElmer, USA) with the excitation wave length of 740 nm and emission wave length of 780 nm. Afterwards, mouse models were sacrificed and perfused with 4% paraformaldehyde. Tumor xenografts were harvested and the semi-quantitative results of fluorescence intensity were also acquired *ex vivo* under the same imaging system.

### *In vivo* distribution of nanomedicines with different size

When three weeks of IMA treatment ended, tumor xenograft-bearing mice received i.v. injection of the mixture of coumarin-6-labeled micelles (Micelles-cou-6) and DiD-labeled NPs (NPs-DiD) at the dose of coumarin-6 and DiD 0.05 mg/kg. Mouse models were sacrificed and subjected to heart perfusion with saline to remove nanomedicines in the circulation system 24 h post nanomedicine injection. Tumor xenografts were then collected and sliced for CD31 labeling as described above. The *in vivo* distribution of Micelles-cou-6 and NPs-DiD within tumor tissues after tumor microenvironment modification was analyzed under a confocal microscope (ZEISS, 710, LSM, Germany) and compared with that of control group.

### Anti-tumor efficacy study

After two-week oral IMA treatment at the daily dose 50 mg/kg, mouse models in the IMA treatment or the control group were then further randomly divided into two groups to receive PTX-loaded micelles (Micelles-PTX) injections or equal volume of saline. The Micelles-PTX treatment was continued every three days for five times (Day 0, Day 3, Day 6, Day 9, Day 12) at the dose of PTX 5 mg/kg. The day of initiating Micelles-PTX treatment was recorded as Day 0. Oral treatment with IMA or deionized water was continued for another one week. Body weight of mouse models and the tumor size were monitored every three days until Day 15. When the entire experiment ended, tumor xenografts were harvested, weighted, and imaged. Afterwards, tumor xenografts were fixed with 4% paraformaldehyde, sliced for H&E staining according to the routine protocols, and examined under the fluorescence microscope (Leica DMI 4000B, Germany). The growth curve of tumor xenografts was also drawn and tumor growth inhibition rate (*TGIR*) was calculated to analyze the therapeutic benefits. *TGIR* based on tumor volume (*TGIR_v_*) and tumor weight (*TGIR_w_*) were calculated by the formulas as following: $\text{TGIRv}=\frac{v_{c}-v_{t}}{{\text{v}}_{c}},\text{TGIRw}=\frac{w_{c}-w_{t}}{w_{c}}$. In these formulas, *V_c_* and *V_t_* represented the tumor volume in the control group and that in the treatment group, respectively; *W_c_* and *W_t_* represented the tumor weight in the control group and that in the treatment group, respectively.

### Statistical analysis

All data were displayed as mean ± SD (standard deviation). Statistical differences were analyzed with unpaired Student's *t*-test for two groups' comparison and one-way ANOVA analysis for multiple-group comparison. *P* value less than 0.05 was considered as statistically significant.

## CONCLUSIONS

In the present study, it was the first time that both nanomedicine size and the tumor microenvironment were optimized simultaneously to achieve an ideal therapeutic benefit. IMA treatment normalized tumor microenvironment including PDGFR-β expression inhibition, tumor vessel normalization and tumor perfusion improvement, which enhanced the *in vivo* delivery of micelles around 23 nm but compromised that of nanoparticles around 110 nm. Furthermore, PTX-loaded smaller micelles achieved the most significant inhibition of tumor growth when pretreated with IMA. Therefore, the present study provided important implications for the rational design of nanomedicine delivery strategy for tumor treatment. In addition, as IMA is now a widely and safely used in clinics, the strategy has great potential to be translated to clinics for tumor treatment.

## SUPPLEMENTARY DATA


