# Supplementary material for: Optimization of the tumor microenvironment and nanomedicine properties simultaneously to improve tumor therapy
Source: Oncotarget. 2016 Aug 23;7(38):62607–18. doi: 10.18632/oncotarget.11546 (PMC5308750; doi:10.18632/oncotarget.11546)
Supplement: Supplementary file 1 [file oncotarget-07-62607-s001.pdf]

Optimization of the tumor microenvironment and nanomedicine properties simultaneously to improve tumor therapy

SUPPLEMENTARY DATA

Adverse effects of IMA treatment

Mouse models were orally given IMA at the dose of 50 mg/kg for three weeks and those treated with deionized water of equal volume served as control. To initially assess the potential adverse effects or tumor growth inhibition effect of IMA treatment in the present study, tumor size and body weight of mouse models were monitored every three days during the whole experiment.

Ex vivo imaging

After three weeks of IMA treatment, mice models were i.v. injected with DiR-labeled NPs (NPs-DiR) or micelles (Micelles-DiR) at the DiR dose of 0.5 mg/kg. 24 h later, mouse models were sacrificed and perfused with 4% paraformaldehyde and major organs including livers, spleens, kidneys, hearts, lungs and brains were harvested and the semi-quantitative results of fluorescence intensity was acquired *ex vivo* under the *In Vivo* IVIS spectrum imaging system (PerkinElmer, USA) with the excitation wave length of 740 nm and emission wave length of 780 nm.

Releasing behavior of PTX-loaded nanomedicine

By using PBS (pH=7.4, 0.01 M) with 0.5% Tween-80 as the release medium, the dialysis bags containing 1 mg PTX-loaded nanomedicines in 1 mL of release medium were incubated in 10 mL of the same medium of at 37 °C with shaking at 100 rpm/min. After sampling at predetermined time points, equal volumes of fresh release medium were added. The concentration of PTX was quantitative analyzed by a HPLC method.

In vivo imaging

After two weeks of IMA treatment ended, mice models were i.v. administrated with DiR-labeled NPs (NPs-DiR) or micelles (Micelles-DiR) at the DiR dose of 0.5 mg/kg. 24 h later, mouse models were subjected to *in vivo* imaging and then sacrificed and perfused with 4% paraformaldehyde. Tumor xenografts were harvested and the semi-quantitative results of fluorescence intensity were acquired *ex vivo* under the same imaging system.

Characterization of Micelles-PTX

Particle size, PDI and zeta potential of PTX-loaded micelles were analyzed using a Malvern Nano ZS (Malvern Instruments, UK) and compared with that of blank micelles.

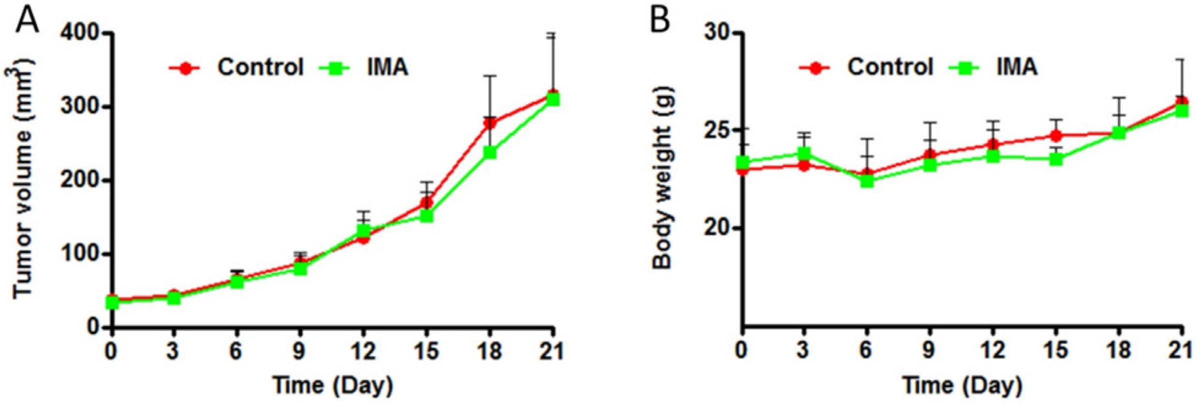

Supplementary Figure S1: The change curves of tumor size A. and body weight **B.** of A549 tumor xenograft-bearing mouse models during the three weeks of IMA or water treatment (n=6). The dose of IMA was 50 mg/kg/d.

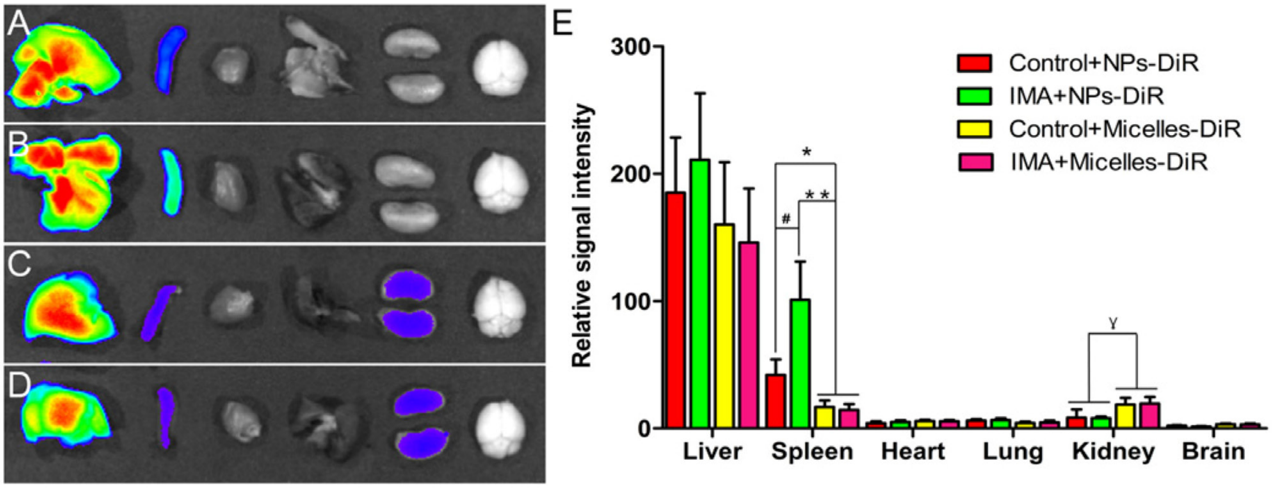

Supplementary Figure S2: *Ex vivo* imaging of major organs (from left to right: liver, spleen, heart, lung, kidney, brain) harvested from deionized water A and C. or IMA **B** and **D.** -treated mouse models 24 h post i.v. injection of NPs-DiR (A and B) or micelles-DiR (C and D) and the corresponding semi-quantitative data **E.** acquired by the *in vivo* imaging system. (A) Control+NPs-DiR group, (B) IMA+NPs-DiR group, (C) Control+Micelles-DiR group, (D) IMA+Micelles-DiR group. $^{*}p<0.05$, compared with Control+NPs-DiR group. $^{**}p<0.01$, compared with IMA+NPs-DiR group. $^{\#}p<0.01$ compared with Control+NPs-DiR group. $^{¥}p<0.05$, compared with Control+NPs-DiR group or IMA+NPs-DiR group.

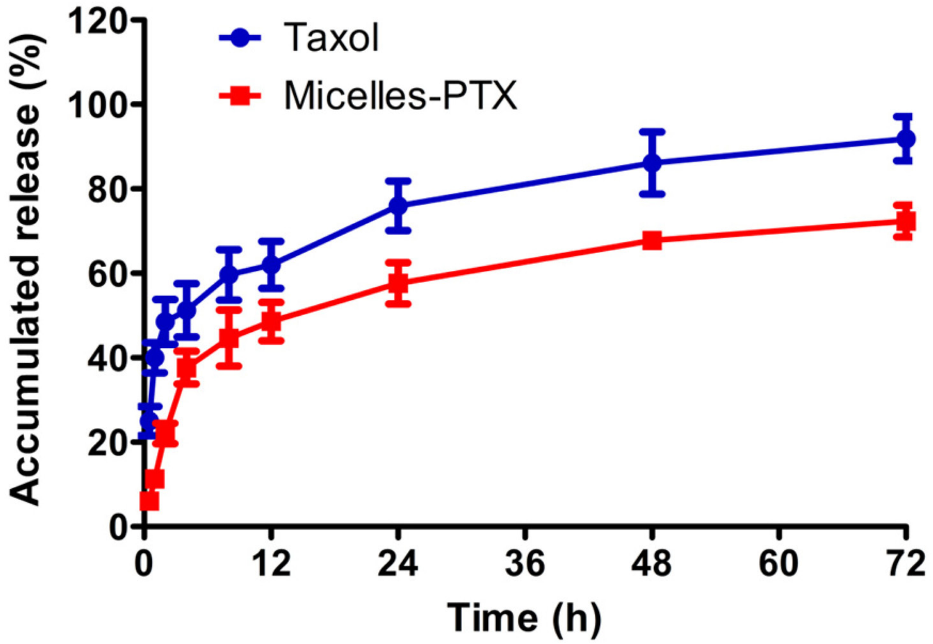

Supplementary Figure S3: The releasing curve of PTX from Taxol and Micelles-PTX in PBS (pH=7.4, 0.01 M) with 0.5% Tween-80 at 37°C.

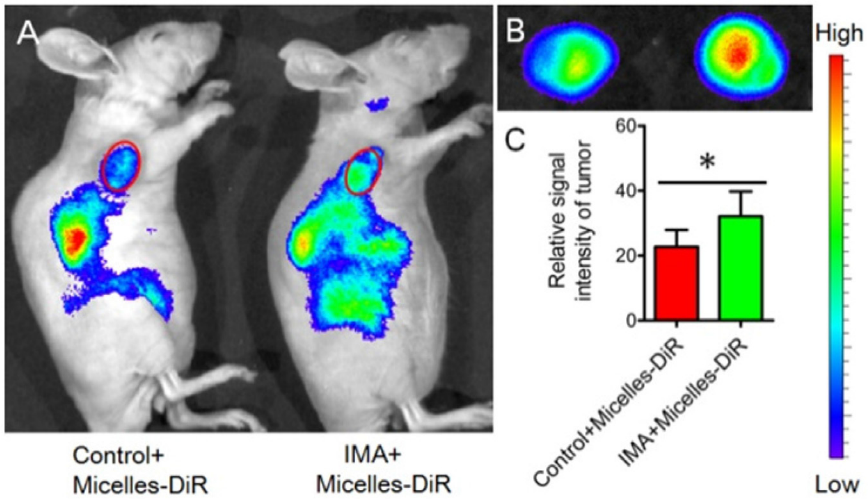

Supplementary Figure S4: The effect of two-week IMA treatment on tumor nanomedicine delivery. After the mouse models were treated with IMA at the daily dose of 50 mg/kg for two weeks, the A549 tumor xenografts were injected with Micelles-DiR and then subjected to *In vivo* imaging. **A.** *In vivo* imaging of mouse models 24 h post Micelles-DiR injection (The dose of DiR was 0.5 mg/kg). **B.** *Ex vivo* imaging of A549 tumor xenografts treated with IMA (right) or not (left) and **C.** the corresponding semi-quantitative fluorescence intensity of A549 tumor xenografts acquired by the *in vivo* imaging system. *P<0.05. Statistical differences were analyzed with unpaired Student's t-test for two groups' comparison.

Supplementary Table S1: Characterization of Micelles-PTX

Group	Size (nm)	PDI	Zeta potential (mV)
Micelles	23.0 ± 1.3	0.07 ± 0.01	-3.1 ± 0.7
Micelles-PTX	25.5 ± 2.6	0.11 ± 0.02	-4.3 ± 0.1
